# Confocal reflectance microscopy in basal cell carcinoma associated with nevus sebaceous: case report^☆^

**DOI:** 10.1016/j.abd.2023.09.011

**Published:** 2024-08-23

**Authors:** Ingrid Priscila Ribeiro Paes Ferraz, Gustavo Carvalho, Juliana Casagrande Tavoloni Braga, Rafaela Brito de Paula, André Molina

**Affiliations:** a Emergency Department, Hospital A.C. Camargo Cancer Center, São Paulo, SP, Brazil; b Department of Cutaneous Oncology, Hospital A.C. Camargo Cancer Center, São Paulo, SP, Brazil

Dear Editor,

Nevus sebaceous is a congenital benign hamartoma of the skin. Its most common complication is transformation into other tumors, usually benign.^1^, ^2^ However, due to the potential for malignancy, early diagnosis and treatment are essential.^3^, ^4^

While dermoscopy allows the analysis of the epidermis to the mid-dermis, reflectance confocal microscopy (RCM) uses an 830-nm diode laser as a monochromatic and coherent light source which penetrates, between 200 and 300 μm, providing images at the cellular level that resemble histopathology, offering detailed morphological analysis of the different skin layers up to the papillary dermis.^5^

There are few reports in the literature regarding typical findings of nevus sebaceous in RCM.^6^, ^7^, ^8^ Articles describing lesions associated with basal cell carcinoma are even less frequent.^9^, ^10^ The authors present a scenario of common dermoscopic characteristics in nevus sebaceous associated with basal cell carcinoma, highlighting changes in confocal reflectance microscopy of the nevus sebaceous, scarcely described in the literature to date.

A 41-year-old male patient with no personal or family history of skin cancer, was treated for a lesion present since childhood on the right forehead with changes in texture and slow growth over the years.

Clinically there was a pearly-yellow plaque, with unclear borders, on an erythematous base and telangiectasias on the periphery, and yellowish papules in its upper region. Palpation showed a slightly verrucous texture (Fig. 1A).

**Figure 1:**
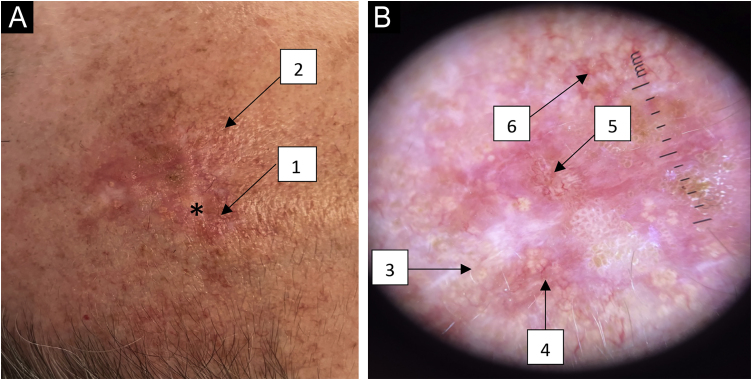
(A) Macroscopy of a pearly-yellow plaque with unclear borders on an erythematous base (*) with telangiectasias on the periphery (1) and yellowish papules (2) on the upper region. (B) Dermoscopy (×10 magnification) showing round whitish-yellow structures in a cobblestone pattern (3), telangiectasias (4), arboriform vessels (5) and rounded whitish-yellow papules with central umbilication and crown vessels (6).

Dermoscopy showed, in the lower region, round and oval, whitish and whitish-yellow uniformly aggregated structures in a cobblestones pattern, with telangiectasias on the periphery. There were arboriform vessels in the central region, which are typically associated with basal cell carcinoma. The upper region showed a group of rounded whitish-yellow papules with central umbilication and crown vessels (Fig. 1B).

RCM, carried out with VivaScope® 1500 (Lucid Inc. Rochester, NY, USA) showed, in the dermis, typical findings of basal cell carcinoma: tumor islands with peritumoral clefting, dark silhouettes and, on the periphery, palisaded cells and dilated tortuous vessels (Fig. 2). At the dermal-epidermal junction and papillary dermis, central tube-shaped structures stood out, with sebaceous gland lobes in the surrounding area, filled with aggregates similar to fish ova, typical of nevus sebaceous (Fig. 3).

**Figure 2:**
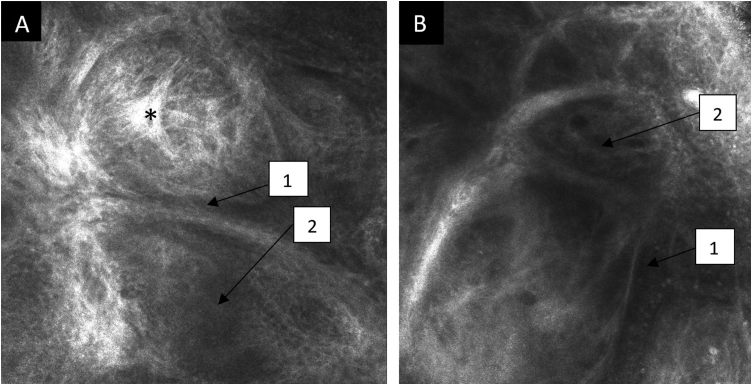
Confocal reflectance microscopy of the papillary dermis showing basal cell carcinoma with individual images measuring 0.5×0.5 mm^2^ and a mosaic measuring 8×8 mm^2^. (A and B) Tumor island (*) with peritumoral clefting (1) and dark silhouettes (2).

**Figure 3:**
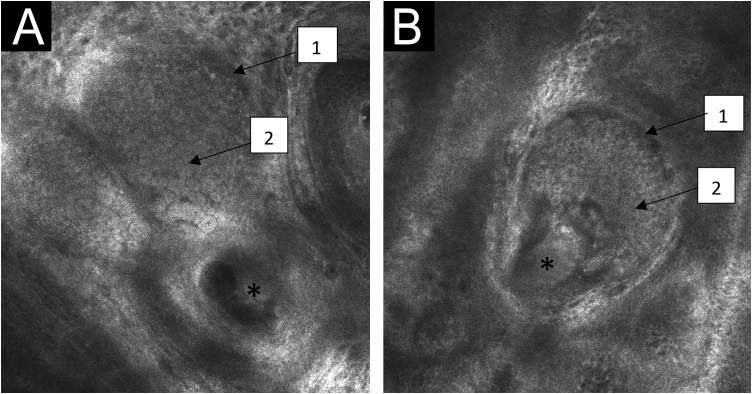
Confocal reflectance microscopy of the papillary dermis of the nevus sebaceous with individual images measuring 0.5×0.5 mm^2^ and mosaics measuring 8×8 mm^2^. (A and B) Central tube-shaped structures (*), with sebaceous gland lobes in the surrounding area (1), filled with aggregates similar to fish ova (2).

Two incisional biopsies were performed with a 4 mm punch. Histopathology revealed in the lower region, superficial basal cell carcinoma and, in the central region, superficial and nodular basal cell carcinoma, both associated with nevus sebaceous (Fig. 4).

**Figure 4:**
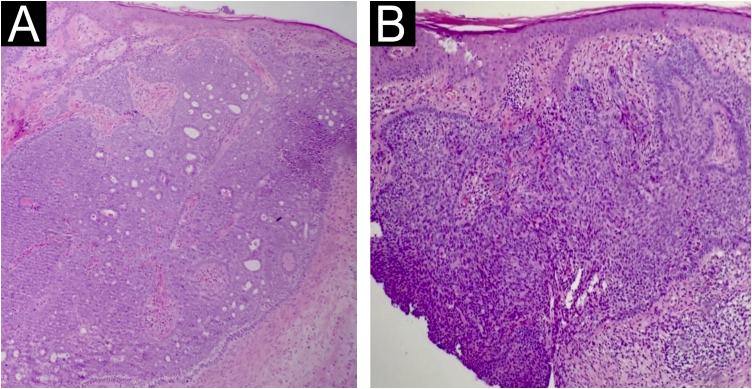
Histopathology. (A) Nodular basal cell carcinoma with an adenoid pattern, in association with a nevus sebaceous. (B) Superficial and nodular basal cell carcinoma in association with a nevus sebaceous (Hematoxylin & eosin, A ×100 and B ×200).

The patient underwent surgery with margin control and primary closure. He has been followed in the Cutaneous Oncology service for six months, with no signs of recurrence.

Nevus sebaceous is a congenital benign hamartoma of the skin consisting of numerous malformed sebaceous glands, degenerated hair follicles and ectopic apocrine glands, most often located on the face and scalp.^1^ Two-thirds of the lesions are present from birth and one-third develop in early childhood.^2^ Its most common complication is transformation into other tumors, more frequently benign ones, firstly trichoblastoma, followed by papillary syringocystadenoma.^3^ Among malignant tumors, the most common is basal cell carcinoma, which develops in less than 1% of cases.^4^ Due to the potential for malignant transformation, early diagnosis and treatment are essential.

While dermoscopy allows the analysis of the epidermis up to the superficial reticular dermis, reflectance confocal microscopy (RCM) uses an 830-nm diode laser as a monochromatic and coherent light source. The penetration depth, between 200 and 300 μm, provides images at the cellular level that resemble histopathology, offering a detailed morphological analysis of the different skin layers up to the papillary dermis.^5^

There are few reports in the literature to date regarding the typical findings of nevus sebaceous in RCM. Descriptions approximate those of sebaceous hyperplasia in RCM.^6^, ^7^, ^8^ A single study identified changes in nevus sebaceus on confocal imaging in different age groups.^9^ The article showed that, under the age of ten, hypoplastic sebaceous glands and juvenile hair follicles can be seen in these lesions. From ten to 59 years, the sebaceous glands at the dermal-epidermal junction resemble bunches of grapes and, in the superficial dermis, structures similar to tubes or loops can be seen in the center, which correspond to the dilation of the sebaceous duct,^7^ with sebaceous gland lobes resembling fish ova in the surrounding area and verrucous or papillomatous hyperplasia in the dermis. Above the age of 60, papillomatous hyperplasia predominates on RCM examination.^1^, ^2^, ^3^

RCM reports describing the characteristics of nevus sebaceous associated with those of basal cell carcinoma, are even less frequent at present, with a single case report of papillary syringocystadenoma and basal cell carcinoma arising from a previous nevus sebaceous.^10^

Therefore, new studies are necessary so that more typical structures of nevus sebaceous associated with basal cell carcinoma are described on RCM.

## Financial support

None declared.

## Authors’ contributions

Ingrid Priscila Ribeiro Paes Ferraz: Approval of the final version of the manuscript; drafting and editing of the manuscript; review of the literature; critical review of the manuscript.

Gustavo Carvalho: Approval of the final version of the manuscript; drafting and editing of the manuscript; review of the literature; critical review of the manuscript.

Juliana Casagrande Tavoloni Braga: Approval of the final version of the manuscript; drafting and editing of the manuscript; review of the literature; critical review of the manuscript.

Rafaela Brito de Paula: Approval of the final version of the manuscript; drafting and editing of the manuscript; review of the literature; critical review of the manuscript.

André Molina: Approval of the final version of the manuscript; drafting and editing of the manuscript; review of the literature; critical review of the manuscript.

## Conflicts of interest

None declared.
